# *IL18* Gene Polymorphism Is Associated with Total IgE in Adult Subjects with Asthma

**DOI:** 10.3390/jcm12123963

**Published:** 2023-06-10

**Authors:** Valentina Lando, Lucia Calciano, Cosetta Minelli, Cristina Bombieri, Marcello Ferrari, Giovanni Malerba, Antonino Margagliotti, Nicola Murgia, Morena Nicolis, Mario Olivieri, James Potts, Stefano Tardivo, Simone Accordini

**Affiliations:** 1 Unit of Epidemiology and Medical Statistics, Department of Diagnostics and Public Health, University of Verona, 37134 Verona, Italysimone.accordini@univr.it (S.A.); 2 National Heart and Lung Institute, Imperial College London, London SW3 6LR, UK; 3 Biology and Genetics Section, Department of Neuroscience, Biomedicine and Movement, University of Verona, 37134 Verona, Italy; 4 Respiratory Diseases Section, Department of Medicine, University of Verona, 37134 Verona, Italy; 5 Department of Environmental and Prevention Sciences, University of Ferrara, 44121 Ferrara, Italy; 6 Unit of Hygiene and Preventive, Environmental and Occupational Medicine, Department of Diagnostics and Public Health, University of Verona, 37134 Verona, Italy; 7 Unit of Occupational Medicine, Department of Diagnostics and Public Health, University of Verona, 37134 Verona, Italy

**Keywords:** asthma, biomarker, *IL18*, polymorphism, SNP, total IgE

## Abstract

The allergic asthma phenotype is characterized by a T helper type 2 (Th2) immune response, based on Immunoglobulin E (IgE)-mediated type 1 hypersensitivity reactions. Total IgE is the sum of all IgE types produced by the human body and is used as a biomarker of inflammation in asthma. We analysed data collected in 143 asthma cases (median age 42.1 years) from the general Italian population (GEIRD survey; 2008–2010) to identify single nucleotide polymorphisms (SNPs) in candidate genes that are associated with total IgE in adult subjects with asthma. These patients reported respiratory symptoms in response to perennial allergens and provided data on 166 SNPs tagging 50 candidate genes or gene regions. Replication of the statistically significant results was performed in 842 asthma cases from other European countries (ECRHS II survey; 1998–2002). SNP rs549908 in interleukin 18 (*IL18)* gene was significantly associated with total IgE in GEIRD, and this result was replicated in ECRHS II. SNP rs1063320 in the human leukocyte antigen G (*HLA-G*) gene was identified in GEIRD, but this association was not replicated in ECRHS II. Further investigating *IL18* and its biological pathways could be important for developing new therapeutic targets, due to its involvement in inflammatory response processes.

## 1. Introduction

Asthma is a multifactorial respiratory disease that depends on the interaction among multiple genetic, environmental, and lifestyle factors [1] and is strongly linked to atopy, allergy, and the response to corticosteroids [2,3]. The allergic asthma phenotype is characterized by a T helper type 2 (Th2) immune response, based on Immunoglobulin E (IgE)-mediated type 1 hypersensitivity reactions [4,5].

IgE belongs to the Ig family, which includes proteins that bind to pathogens and harmful substances (bacteria, viruses, and allergens) to protect the human body [4]. IgE antibodies are produced by activated B cells in response to interleukin (IL) 4 and IL13 cytokines, released by activated Th2 cells. IgE antibodies are released into the circulation and bind effector cells through their receptors [6]. Upon re-exposure to an allergen, the antigen-IgE complex triggers the release of pro-inflammatory mediators (histamine, leukotrienes, and cytokines) by eosinophils, basophils, and mast cells, resulting in local pathophysiological events (such as bronchoconstriction, vasodilation, and airway mucus secretion) that are responsible for triggering respiratory symptoms [5,6,7,8]. Total IgE is the sum of all IgE types that are produced by the human body and is used as a biomarker of asthma inflammation [9,10], with higher levels during childhood and in male individuals [11,12]. Tobacco smoking [13], alcohol consumption [14], and the immunological status of patients [15] also increase total IgE. Associations between total IgE and single nucleotide polymorphisms (SNPs) in some genes, such as Fc epsilon receptor 1A (*FCER1A*), *IL13*, signal transducer and activator of transcription 6 (*STAT6*), cytotoxic T lymphocyte associated protein 4 (*CTLA4*), and A disintegrin and metalloproteinase domain 33 (*ADAM33*), have previously been described [16,17,18,19,20,21]. Variants in these genes can lead to dysregulations in the IgE production pathway.

The aim of this study is to identify SNPs in candidate genes associated with total IgE in adult subjects with asthma. This genetic association analysis was performed using Italian data from the Gene Environment Interactions in Respiratory Diseases (GEIRD) study on SNPs in candidate genes [22]. Replication of statistically significant results was carried out within the European Community Respiratory Health Survey (ECRHS) II (www.ecrhs.org (accessed on 18 May 2023)) [23,24].

## 2. Materials and Methods

### 2.1. The GEIRD Survey

**Figure 1:**
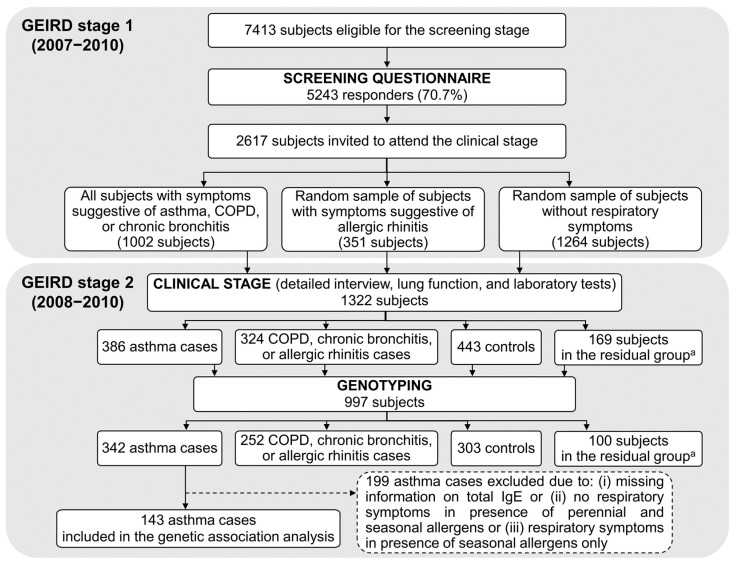
Selection of the asthma cases included in the genetic association analysis (GEIRD survey; Verona centre). GEIRD: Gene Environment Interactions in Respiratory Diseases; COPD: chronic obstructive pulmonary disease; IgE: Immunoglobulin E. ^a^ Subjects who did not fulfil the criteria for cases or controls.

GEIRD is a candidate gene-based, (multi)case-control study on the role of genetic and modifiable factors in respiratory diseases (asthma, chronic obstructive pulmonary disease (COPD), chronic bronchitis, and allergic rhinitis) in adults. The protocol is described elsewhere [22]. Briefly, cases and controls were identified through a two-stage process in pre-existing cohorts [23,24,25] and in new random samples of the Italian general population [22]. Stage 1 (2007–2010) was the screening phase, in which 7413 subjects from the Verona centre received a respiratory health screening questionnaire by mail (response rate 70.7%; Figure 1). Stage 2 (2008–2010) was the clinical phase, in which 2617 responders to the screening questionnaire in Verona underwent a detailed interview, spirometry, and laboratory tests for identifying cases and controls.

Blood samples were collected and stored for IgE titration and genomic DNA extraction according to standardised international protocols [22]. Total IgE levels were measured using the Pharmacia CAP system (Uppsala, Sweden) and were expressed as kiloUnits per Litre (kU/L). Three hundred and eighty-four SNPs tagging 53 candidate genes or gene regions (listed in Table S1) were evaluated in the original survey (2008–2010) [26]. Gene selection in GEIRD is described in the online Supplementary Materials. GoldenGate Genotyping assay (Illumina) was used for genotyping polymorphisms.

In Verona, 1322 cases and controls were identified in the clinical stage, and 997 of these individuals were genotyped. The GEIRD study in this centre was approved by the local ethics committee (“Comitato Etico per la Sperimentazione dell’Azienda Ospedaliera Istituti Ospitalieri di Verona”), and the participants gave their written informed consent after a full explanation of all the aspects of the research project.

### 2.2. Identification of Cases and Controls in GEIRD

Asthma cases were the subjects who met at least one of the following criteria [22]:

1. having reported asthma at any time;
2. having reported asthma-like symptoms (asthma attacks, wheezing, chest tightness, shortness of breath (SoB) at rest, SoB at night-time, SoB following strenuous activities) or the utilization of anti-asthmatic drugs in the previous 12 months, and having at least one of the following clinical characteristics:
  - Positive methacholine challenge test (provocative dose (PD_20_) causing a 20% fall in forced expiratory volume in one second (FEV_1_) < 1 mg);
  - Pre-bronchodilator (BD) airflow obstruction (AO) (FEV_1_/forced vital capacity (FVC) <lower limit of normal (LLN) [27] or <70%) and a positive reversibility test (increase in post-BD FEV_1_ > 12% and >200 mL with respect to pre-BD FEV_1_ after 400 mcg of salbutamol);
  - Pre- but not post-BD AO, and post-BD FEV_1_ ≥ 80% predicted.

The definition of COPD, chronic bronchitis, or allergic rhinitis is reported elsewhere [22]. Controls were the subjects who did not fulfil the criteria for case identification and who had pre-BD FEV_1_ > 70% predicted and no pre-BD AO. A residual group included the individuals who were neither cases nor controls.

### 2.3. Study Subjects

Genetic data were only available for participants in GEIRD stage 2 from Verona. The study subjects were 143 asthma cases (out of 342 genotyped patients) with total IgE measurement, who had reported respiratory symptoms in response to perennial allergens and, eventually, also to seasonal allergens (Figure 1). Data from all genotyped subjects were used for additional SNP quality checks.

### 2.4. Genetic Association Analysis

In this analysis, we considered all the SNPs available in the GEIRD dataset (384 polymorphisms tagging 53 genes or gene regions) and, among these SNPs, we analysed the 166 polymorphisms in 50 genes or gene regions (listed in Table S1) that satisfied the following additional quality checks:

1. Genotype failure rate ≤ 5% in all genotyped subjects (n = 997);
2. Genotype failure rate ≤ 5% in all asthma cases (n = 342);
3. Minimum genotype frequency ≥5% in all asthma cases (n = 342);
4. Allele frequencies needed to respect Hardy–Weinberg equilibrium (HWE) in the controls (n = 303). The SNPs that were not available in the control group were excluded from the analysis [28]. *p*-values for testing deviation from HWE were corrected for the False Discovery Rate (FDR) using the Benjamini–Yekutieli procedure [29];
5. Linkage disequilibrium (LD; squared correlation between allelic values at two loci (*r*^2^) < 0.8) in the asthma cases included in the study (n = 143) to avoid redundant testing. In case of *r*^2^ ≥ 0.8, the SNP with the highest sample size for the homozygous genotype with lower allele frequency was selected for the analysis.

The association with total IgE was assessed separately for each polymorphism using a quasi-gamma log-link regression model [30]. All regression models had total IgE as the outcome and the SNP (classified according to the additive genetic model, with the homozygous genotype with higher allele frequency as the reference), age, and sex as covariates. The strength of the association was measured through the ratio of expected total IgE between the heterozygous genotype (or the homozygous genotype with lower allele frequency) and the reference. The iterated, reweighted least-squares optimization of the deviance was used to obtain the maximum likelihood estimates, taking extra variability into account.

### 2.5. Replication Analysis

SNPs identified in GEIRD were tested for replication in an independent sample of 842 asthma cases who had participated in the ECRHS II survey (from 16 centres located in Estonia, France, Germany, Norway, Spain, Sweden, Switzerland, and United Kingdom). Briefly, ECRHS is an international, population-based cohort study of respiratory health in subjects aged 20–44 years at the time of recruitment (ECRHS I; 1991–1993) [23]. Each participant was mailed a screening questionnaire (stage 1) and a 20% “random sample” of the responders was invited to undergo a detailed clinical examination (stage 2). An additional “symptomatic sample” of subjects with asthma-like symptoms was recruited at stage 2. The participants in ECRHS I stage 2 were re-examined in 1998–2002 (ECRHS II) [24]. Asthma cases were identified according to comparable phenotype criteria (see the online Supplementary Materials) [24] with those used in GEIRD and provided blood samples for genotyping and IgE titration at ECRHS II. Total IgE levels were measured using the Pharmacia CAP system (Uppsala, Sweden) and expressed as kiloUnits per Litre (kU/L). The genotyping method used to analyse the SNPs was the SNPlexTM platform (Applied Biosystems), used according to the manufacturer’s instructions [31].

The association between each SNP (identified in GEIRD) and total IgE was evaluated by a 2-level (subject: level 1 unit; centre: level 2 unit) quasi-gamma log-link regression model, with age and sex as adjustment variables. Multilevel modelling was carried out to take the hierarchical structure of ECRHS II data into account. Replication was defined as an effect estimate in the same direction as in GEIRD and, therefore, one-sided p-values were calculated. Replication p-values were corrected to control the FDR using the Simes procedure [32].

Two sensitivity analyses were carried out for the SNPs that were found to be significantly associated with total IgE after FDR correction in the replication set. The first sensitivity analysis was performed within a restricted ECRHS II sample of 51 asthma cases with respiratory symptoms only in the presence of perennial allergens, to exclude an effect due to seasonal exposure. The second sensitivity analysis was performed within the GEIRD control group (n = 288) to test whether the replicated SNPs were associated with total IgE independently of asthma.

All statistical analyses were carried out using STATA software (release 17; StataCorp, College Station, TX, USA), with the exception of the additional SNP quality checks that were performed using R software (version 4.1.2; The R Foundation for Statistical Computing, Vienna, Austria; “HardyWeinberg” package, cran.r-project.org/web/packages/HardyWeinberg).

## 3. Results

### 3.1. Main Characteristics of the Asthma Cases

The 143 asthma cases (female 49.7%, past smoker 22.4%, current smoker 28.7%) who were identified in GEIRD and were included in the genetic association analysis had a median age of 42.1 years and a median BMI of 24.5 (Table 1).

Of these patients, 69.2% had nasal allergies, 34.3% had eczema/other skin allergies, and 14.0% had an itchy rash. The median pre-BD FEV_1_ % predicted was 95.5, the median FVC % predicted was 101.7, and the median FEV_1_/FVC % predicted was 93.3. The geometric mean of total IgE was 111.5 kU/L. A statistically significant difference in the percentage of asthma cases with nasal allergies (*p* < 0.001) and in the geometric mean of total IgE (*p* = 0.036) was observed in GEIRD between the study subjects and the 243 eligible patients excluded from the analysis (Table S3). These cases were not evaluated in our study because they had not been genotyped (44 subjects) or, among those with genetic data, because (i) they lacked information on total IgE, (ii) they had reported no respiratory symptoms in the presence of perennial and seasonal allergens, or (iii) they had reported respiratory symptoms in the presence of seasonal allergens only (199 subjects) (Figure 1).

Compared to the 143 study patients (GEIRD dataset), the 842 asthma cases who were assessed in the replication analysis (ECRHS II dataset) had higher BMI (median: 25.1 vs. 24.5, *p* = 0.024), a lower percentage of subjects with nasal allergies (44.1% vs. 69.2%, *p* < 0.001), a higher percentage of subjects with eczema/other skin allergies (51.8% vs. 34.3%, *p* < 0.001), a higher percentage of subjects reporting an itchy rash (23.2% vs. 14.0%, *p* = 0.013), and lower total IgE (geometric mean: 54.1 vs. 111.5, *p* = 0.003) (Table 1).

### 3.2. Genetic Association and Replication Analyses

Thirteen polymorphisms were significantly associated with total IgE in GEIRD (Table 2): rs898070 (*NPSR1*; *p* < 0.001), rs12830203 (*NOS1*; *p* = 0.004), rs2072862 (*IL2RB*; *p* = 0.004), rs11635145 (*SMAD3A*; *p* = 0.005), rs6817700 (*NPNT*; *p* = 0.006), rs733334 (*NOS1*; *p* = 0.008), rs10184597 (*IL1RL2*; *p* = 0.009), rs549908 (*IL18*; *p* = 0.016), rs9389986 (*GPR126*; *p* = 0.023), rs4760648 (*VDR*; *p* = 0.032), rs578776 (*CHRNA3*; *p* = 0.042), rs3791978 (*TNS1*; *p* = 0.048), and rs1063320 (*HLA-G*; *p* = 0.048).

The observed association of SNP rs549908 in *IL18* gene with total IgE was replicated in ECRHS II (uncorrected one-sided p-value, *p* = 0.004; FDR-corrected one-sided *p*-value, *p* = 0.054). The result for SNP rs1063320 in human leukocyte antigen G (*HLA-G*) gene did not reach statistical significance after correcting for multiple testing (uncorrected one-sided p-value, *p* = 0.031; FDR-corrected one-sided p-value, *p* = 0.198) (Table 2).

The main characteristics of the GEIRD patients according to rs549908 genotypes (TT, n = 73; TG, n = 60; GG, n = 9) are described in Table 3. The three groups of asthma cases were similar according to the distribution of all variables apart from pre-BD FEV_1_ % predicted (*p* = 0.034) and total IgE (*p* = 0.005). The expected value of total IgE (obtained with a quasi-gamma log-link regression model, which included the SNP as a qualitative variable and age and sex as adjustment variables) was 154.5 (95% confidence interval, 95%CI: 116.5, 192.6), 270.7 (196.8, 344.6), and 174.7 (51.6, 297.7) kU/L among the GEIRD patients with TT, TG, or GG genotypes, respectively.

### 3.3. Sensitivity Analyses

The estimated effect of SNP rs549908 (*IL18*) within the ECRHS II restricted sample (ratio of expected total IgE (95%CI): 1.48 (0.95, 2.31)) was in the same direction as that obtained within the whole replication set (Table 2). This result supports the hypothesis that the association observed in the replication sample is not due to a seasonal exposure effect, although statistical significance was not reached due to the small sample size of the restricted group (n = 51). No association was found between this polymorphism and total IgE within the control group in the GEIRD dataset, suggesting that this result appears to be dependent on the disease state of the subjects.

## 4. Discussion

The main results of the study are the following:

- SNP rs549908 in the *IL18* gene was associated with total IgE in two independent samples (GEIRD and ECRHS II) of adult subjects with asthma from the general European population;
- SNP rs1063320 in the *HLA-G* gene was linked with this biomarker of inflammation, although statistical significance was not maintained after correcting for multiple testing in the replication sample (ECRHS II).

In this analysis, the effect allele G of the synonymous variant rs549908 was associated with higher levels of total IgE in both the GEIRD and ECRHS II datasets. Despite the fact that the association between SNP rs549908 and total IgE in asthma or other allergic respiratory conditions has not been evaluated in Caucasians, statistically significant results were obtained in this population for polymorphisms in LD with rs549908 (rs1946519 (*r^2^* = 0.524), rs1946518 (*r*^2^ = 0.524), rs187238 (*r^2^* = 0.842), rs360718 (*r^2^* = 0.842), rs360717 (*r^2^* = 0.842), and rs360721 (*r^2^* = 1.0); *r^2^* was calculated for the population of Northern Europeans from Utah (CEU) using the LDlink web application (ldlink.nih.gov (accessed on 18 May 2023))) [33,34,35]. However, these results are not fully comparable with ours due to differences in the study population and age distribution and have not been replicated in other populations. In addition, other studies have identified SNPs in the *IL18* receptor gene cluster and in the *IL1* family that are linked to asthma-associated biomarkers (i.e., serum IgE, blood eosinophilic counts) in asthma or other allergic disorders [36,37,38,39,40].

The *IL18* gene encodes a pleiotropic pro-inflammatory cytokine that is mainly involved in innate and adaptive immune responses in allergic diseases, such as asthma. Asthma is characterized by a Th2-type airway inflammation with eosinophils, IgE production, and airway hyperresponsiveness [41]. This protein belongs to the IL1 family and is expressed by macrophages, dendritic cells, and epithelial cells, as well as smooth muscle cells [42,43]. Its pleiotropic role is determined by which other cytokines are present in the extracellular environment [43,44,45]. IL18 triggers the synthesis and the subsequent release of vasoactive and inflammatory mediators, such as IL4 and IL13, by basophils and mast cells, which in turn amplify IgE production [46,47]. The production and secretion of Th2 cytokines (IL4 and IL13) by differentiated effector cells can also occur in response to IL18 alone, without stimulation of the IgE-receptor complex [48].

We found that the effect allele G of SNP rs1063320 in the *HLA-G* gene was associated with lower levels of total IgE, although this result did not reach statistical significance after multiple testing correction in the ECRHS II dataset.

The *HLA-G* gene encodes a major histocompatibility complex class I protein, which is expressed in bronchial epithelium cells. Although the role of HLA-G in asthma is unclear, this protein interacts with T, B, and natural killer cell receptors to modulate immune responses by blocking and/or inhibiting their functions in both soluble and membrane-bound isoforms [49,50]. In the literature, two genome-wide association studies have identified the *HLA-G* gene in relation to total IgE [51,52]. Other studies have shown that elevated levels of soluble HLA-G from the bronchoalveolar lavage fluid are associated with low serum IgE [53] or that serum HLA-G expression levels, which are increased in inflammatory diseases (e.g., asthma and allergic rhinitis), are associated with increased serum levels of specific IgE [50,54,55]. Lower levels of HLA-G may be due to the presence of highly polymorphic sites in the 3′ untranslated region, such as rs1063320, that control post-transcriptional *HLA-G* mRNA stability and miRNA regulation [49,56,57,58]. Despite the lack of replication in ECRHS II, this gene represents an interesting target for further investigation of its role in asthma and its involvement in IgE production.

A major strength of this study is the replication analysis conducted on a relatively large group of asthma patients from other European countries. Furthermore, accurate phenotyping was performed in both GEIRD [22] and ECRHS II [24] using similar standardised protocols. Finally, total IgE was analysed as a continuous rather than categorical variable, as there is no clear consensus in the literature on the cut-off to be used to subdivide patients according to total IgE levels [12,59,60]. The use of a continuous outcome should increase the probability of identifying polymorphisms, as the use of an incorrect cut-off could hide true associations.

Some caveats should be taken into account when interpreting our results. The number of asthma cases in the genetic association analysis (GEIRD dataset; n = 143) is relatively small. However, the evaluation of asthma cases from the general population allows a wider range of disease phenotypes to be captured than the evaluation of clinically selected patients. Furthermore, the limitation due to the relatively small sample size was partly overcome by replicating the results in a larger independent group of patients (ECRHS II dataset; n = 842). In addition, GEIRD is a candidate gene study with a limited number of genotyped SNPs. Finally, two cohorts of European ancestry were analysed in this study, which limits the generalisability of our results to other populations due to the influence of ethnicity on total IgE [61].

## 5. Conclusions

We found that SNP rs549908 in the *IL18* gene is associated with total IgE among adult subjects with asthma, selected from the general European population in two independent surveys. Further investigation of *IL18* and its biological pathways could be important for the development of new therapeutic targets, as it is mainly involved in inflammatory response processes. We also identified SNP rs1063320 in the *HLA-G* gene in patients from the general Italian population; this deserves additional examination to better understand its role in the allergic asthma phenotype.

## Figures and Tables

**Table 1:** Main characteristics of the asthma cases in GEIRD (genetic association analysis) and in ECRHS II (replication analysis).

		Genetic Association Analysis	Replication Analysis	*p*-Value ^a,b^
Sample, n		143	842	-
Female, %		49.7	53.9	0.344
Age (years), median (IQR)		42.1 (34.9, 48.5)	42.9 (37.2, 48.8)	0.204
BMI, median (IQR)		24.5 (22.0, 27.1)	25.1 (22.8, 28.1)	0.024
Tobacco smoking, %	Never	48.9	40.3	0.143
	Past	22.4	27.6	
	Current	28.7	32.1	
Nasal allergies, %	Absent	30.1	55.2	<0.001
	Present	69.2	44.1	
	Missing	0.7	0.7	
Eczema/Skin allergies, %		34.3	51.8	<0.001
Itchy rash, %		14.0	23.2	0.013
Pre-BD FEV_1_ % predicted, median (IQR)		95.5 (85.7, 108.3)	97.8 (86.5, 108.4)	0.388
Pre-BD FVC % predicted, median (IQR)		101.7 (92.2, 110.0)	99.2 (88.8, 108.7)	0.130
Pre-BD FEV_1_/FVC % predicted, median (IQR)		93.3 (89.4, 100.4)	95.6 (89.5, 104.3)	0.066
Total IgE (kU/L), geometric mean (95%CI)		111.5 (91.5, 135.8)	54.1 (49.0, 59.7)	0.003

GEIRD: Gene Environment Interactions in Respiratory Diseases; ECRHS: European Community Respiratory Health Survey; IQR: interquartile range; BMI: body mass index; pre-BD: pre-bronchodilator; FEV_1_: forced expiratory volume in one second; FVC: forced vital capacity; IgE: Immunoglobulin E; kU/L: kiloUnits per Litre; 95%CI: 95% confidence interval. ^a^ Pearson chi-squared test, Fisher’s exact test, Wilcoxon rank-sum test, or likelihood-ratio test were used when needed. ^b^ Comparison between the 143 GEIRD subjects included in the genetic association analysis and the 842 ECRHS II subjects included in the replication analysis.

**Table 2:** SNPs identified in GEIRD (genetic association analysis) and tested in ECRHS II (replication analysis).

		Genetic Association Analysis				Replication Analysis					
Gene or Gene Region	SNP	Genotype	Sample (n = 143)	Ratio of Expected Total IgE ^a^ (95%CI)	Two-Sided *p*-Value	Genotype ^b^	Sample (n = 842)	Ratio of Expected Total IgE ^c^ (95%CI)	Two-Sided *p*-Value	One-Sided *p*-Value ^d^	FDR-Corrected One-Sided *p*-Value
*IL18*	rs549908	TT	73	1.00	-	TT	379	1.00	-	-	-
		TG	60	**1.43 (1.07, 1.92)**	**0.016**	TG	375	**1.32 (1.07, 1.62)**	**0.008**	**0.004**	**0.054**
		GG	9	2.05 (additive effect)	-	GG	88	1.74 (additive effect)	-	-	-
*HLA-G*	rs1063320	CC	46	1.00	-	CC	211	1.00	-	-	-
		CG	66	**0.78 (0.61, 0.998)**	**0.048**	CG	413	0.84 (0.71, 1.01)	0.061	**0.031**	0.198
		GG	31	0.60 (additive effect)	-	GG	218	0.71 (additive effect)	-	-	-
*NPSR1*	rs898070	GG	60	1.00	-	GG	329	1.00	-	-	-
		AG	63	**0.61 (0.48, 0.79)**	**0.0001**	AG	409	1.07 (0.75, 1.51)	0.719	0.641	0.757
		AA	19	0.38 (additive effect)	-	AA	104	1.14 (additive effect)		-	-
*NOS1*	rs12830203	CC	53	1.00	-	CC	419	1.00	-	-	-
		TC	75	**0.66 (0.50, 0.87)**	**0.004**	TC	339	1.07 (0.87, 1.31)	0.527	0.736	0.798
		TT	15	0.44 (additive effect)	-	TT	84	1.14 (additive effect)	-	-	-
*IL2RB*	rs2072862	GG	53	1.00	-	GG	403	1.00	-	-	-
		AG	68	**1.47 (1.13, 1.90)**	**0.004**	AG	345	1.11 (0.88, 1.40)	0.362	0.181	0.470
		AA	21	2.16 (additive effect)	-	AA	94	1.24 (additive effect)	-	-	-
*SMAD3A*	rs11635145	AA	46	1.00	-	AA	235	1.00	-	-	-
		AG	72	**1.42 (1.11, 1.82)**	**0.005**	AG	423	0.85 (0.67, 1.08)	0.192	0.904	0.904
		GG	25	2.03 (additive effect)	-	GG	184	0.73 (additive effect)	-	-	-
*NPNT*	rs6817700	GG	37	1.00	-	GG	231	1.00	-	-	-
		AG	79	**1.45 (1.11, 1.89)**	**0.006**	AG	422	1.03 (0.89, 1.18)	0.724	0.362	0.748
		AA	27	2.10 (additive effect)	-	AA	189	1.05 (additive effect)	-	-	-
*NOS1*	rs733334	GG	35	1.00	-	CC	214	1.00	-	-	-
		AG	73	**0.71 (0.55, 0.91)**	**0.008**	TC	427	1.003 (0.83, 1.21)	0.976	0.512	0.748
		AA	35	0.51 (additive effect)	-	TT	201	1.01 (additive effect)	-	-	-
*IL1RL2*	rs10184597	CC	78	1.00	-	CC	437	1.00	-	-	-
		TC	54	**1.47 (1.10, 1.92)**	**0.009**	TC	355	1.09 (0.92, 1.29)	0.331	0.166	0.470
		TT	10	2.17 (additive effect)	-	TT	50	1.18 (additive effect)	-	-	-
*GPR126*	rs9389986	TT	58	1.00	-	TT	390	1.00	-	-	-
		AT	61	**0.74 (0.56, 0.96)**	**0.023**	AT	367	0.98 (0.81, 1.18)	0.814	0.407	0.748
		AA	24	0.54 (additive effect)	-	AA	85	0.96 (additive effect)	-	-	-
*VDR*	rs4760648	CC	43	1.00	-	CC	272	1.00	-	-	-
		TC	68	**0.76 (0.60, 0.98)**	**0.032**	TC	401	0.93 (0.82, 1.05)	0.232	0.116	0.470
		TT	29	0.58 (additive effect)	-	TT	169	0.86 (additive effect)	-	-	-
*CHRNA3*	rs578776	CC	74	1.00	-	GG	443	1.00	-	-	-
		TC	57	**1.36 (1.01, 1.84)**	**0.042**	AG	335	0.97 (0.70, 1.34)	0.851	0.575	0.748
		TT	10	1.86 (additive effect)	-	AA	64	0.94 (additive effect)	-	-	-
*TNS1*	rs3791978	TT	40	1.00	-	AA	236	1.00	-	-	-
		TG	71	**0.77 (0.59, 0.998)**	**0.048**	AC	414	1.01 (0.89, 1.16)	0.850	0.575	0.748
		GG	32	0.59 (additive effect)	-	CC	192	1.03 (additive effect)	-	-	-

SNP: single nucleotide polymorphism; GEIRD: Gene Environment Interactions in Respiratory Diseases; ECRHS: European Community Respiratory Health Survey; IgE: Immunoglobulin E; 95%CI: 95% confidence interval; FDR: false discovery rate; *NPSR1*: neuropeptide S receptor 1; *NOS1*: nitric oxide synthase 1; *IL2RB*: IL 2 receptor subunit beta; *SMAD3A*: SMAD family member 3; *NPNT*: nephronectin; *IL1RL2*: interleukin 1 receptor like 2; *IL18*: interleukin 18; *GPR126*: G protein-coupled receptor 126; *VDR*: vitamin D receptor; *CHRNA3*: cholinergic receptor nicotinic alpha 3 subunit; *TNS1*: tensin 1; *HLA-G*: human leukocyte antigen G. The statistically significant associations are reported in bold. ^a^ Ratio of expected total IgE between the heterozygous and the homozygous genotype with higher allele frequency (reference) for a given SNP (classified according to the additive genetic model), obtained by a quasi-gamma log-link regression model, with sex and age as adjustment variables. ^b^ Identified by alleles reported in the forward orientation; differences between GEIRD and ECRHS II genotypes are due to the strand (forward or reverse) that was sequenced in the two surveys. ^c^ Obtained by a 2-level (subject: level 1 unit; centre: level 2 unit) quasi-gamma log-link regression model, with the SNP (classified according to the additive genetic model), age, and sex as covariates. ^d^ Computed according to the direction of the regression coefficients in GEIRD and ECRHS II.

**Table 3:** Main characteristics of the asthma cases ^a^ according to SNP rs549908 (*IL18*) genotype (GEIRD survey).

		TT (n = 73)	TG (n = 60)	GG (n = 9)	*p*-Value ^b^
Sample, n		52.0	45.0	66.7	0.439
Female, %		42.0 (36.1, 48.3)	42.5 (31.1, 48.1)	44.7 (39.9, 48.5)	0.380
Age (years), median (IQR)		24.5 (22.1, 26.7)	24.4 (21.7, 27.3)	25.5 (23.1, 29.4)	0.693
BMI, median (IQR)		54.8	45.0	22.2	0.278
Tobacco smoking, %	Never	20.6	21.7	44.4	
	Past	24.7	33.3	33.3	
	Current	74.0	66.7	44.4	0.413
Nasal allergies, %	Absent	35.6	23.3	33.3	0.342
	Present	63.0	76.7	66.7	
	Missing	1.4	0.0	0.0	
Eczema/Skin allergies, %		37.0	35.0	11.1	0.344
Itchy rash, %		13.7	16.7	0.0	0.508
Pre-BD FEV_1_ % predicted, median (IQR)		95.5 (84.7, 106.3)	97.8 (90.1, 108.6)	84.0 (70.5, 93.5)	0.034
Pre-BD FVC % predicted, median (IQR)		101.1 (92.2, 109.2)	102.2 (94.7, 110.7)	90.5 (85.7, 101.4)	0.199
Pre-BD FEV_1_/FVC % predicted, median (IQR)		94.1 (90.9, 100.8)	94.1 (90.2, 100.4)	88.2 (81.7, 93.2)	0.059
Total IgE (kU/L), geometric mean (95%CI)		88.4 (68.0, 115.1)	151.8 (110.3, 209.0)	99.9 (41.8, 238.8)	0.005

SNP: single nucleotide polymorphism; IL18: interleukin 18; GEIRD: Gene Environment Interactions in Respiratory Diseases; IQR: interquartile range; BMI: body mass index; pre-BD: pre-bronchodilator; FEV1: forced expiratory volume in one second; FVC: forced vital capacity; IgE: Immunoglobulin E; kU/L: kiloUnits per Litre; 95%CI: 95% confidence interval. ^a^ One of the 143 asthma cases had missing genotypes in this polymorphism. ^b^ Pearson chi-squared test, Fisher’s exact test, Wilcoxon rank-sum test, or likelihood-ratio test were used when needed.

## Data Availability

Due to data protection reasons, the datasets that were analysed in this study cannot be made publicly available.

## Supplementary Materials

The following supporting information can be downloaded at: https://www.mdpi.com/article/10.3390/jcm12123963/s1, Table S1: List of the 384 tag-SNPs in 53 candidate genes or gene regions that were measured in the GEIRD survey (2008–2010). The 166 SNPs included in the genetic association analysis are reported; Paragraph S1: Gene selection in the GEIRD study; Table S2: List of the references that were used to select the 53 genes or gene regions in the GEIRD survey (2008–2010); Paragraph S2: Definition of asthma in the ECRHS II study; Table S3: Main characteristics of the asthma cases according to their inclusion in the genetic association analysis (GEIRD survey); Paragraph S3: Supplementary information on the ECRHS II study.
