# Supplementary material for: IL18 Gene Polymorphism Is Associated with Total IgE in Adult Subjects with Asthma
Source: J Clin Med. 2023 Jun 10;12(12):3963. doi: 10.3390/jcm12123963 (PMC10299306; doi:10.3390/jcm12123963)
Supplement: Supplementary file 1 [file jcm-12-03963-s001.zip › jcm-2433981-supplementary.pdf]

SUPPLEMENTARY MATERIALS

IL18 Gene Polymorphism is Associated with Total IgE in Adult Subjects with Asthma

Valentina Lando[1,*], Lucia Calciano[1], Cosetta Minelli[2], Cristina Bombieri[3], Marcello Ferrari[4], Giovanni Malerba[3], Antonino Margagliotti[1], Nicola Murgia[5], Morena Nicolis[6], Mario Olivieri[7], James Potts[2], Stefano Tardivo[6] and Simone Accordini[1]

[1] Unit of Epidemiology and Medical Statistics, Department of Diagnostics and Public Health, University of Verona, 37134 Verona, Italy
[2] National Heart and Lung Institute, Imperial College London, London SW3 6LR, United Kingdom
[3] Biology and Genetics Section, Department of Neuroscience, Biomedicine and Movement, University of Verona, 37134 Verona, Italy
[4] Respiratory Diseases Section, Department of Medicine, University of Verona, 37134 Verona, Italy (Retired)
[5] Department of Environmental and Prevention Sciences, University of Ferrara, 44121 Ferrara, Italy
[6] Unit of Hygiene and Preventive, Environmental and Occupational Medicine, Department of Diagnostics and Public Health, University of Verona, 37134 Verona, Italy
[7] Unit of Occupational Medicine, Department of Diagnostics and Public Health, University of Verona, 37134 Verona, Italy (Retired)
* Correspondence: valentina.lando@univr.it

Table S1. List of the 384 tag-SNPs in 53 candidate genes or gene regions that were measured in the GEIRD survey (2008-2010). The 166 SNPs included in the genetic association analysis are reported.

SNP	Gene or gene region	Chromosome	Chr Pos (GRCh37.p1 assembly)	Included
rs4147567	*GSTM1*	1	110232524	
rs10857796	*GSTM1*	1	110235032	
rs2247584	*FCER1A*	1	159259680	X
rs2427828	*FCER1A*	1	159273041	X
rs2494265	*FCER1A*	1	159275274	
rs2427829	*FCER1A*	1	159282011	X
rs2275352	*CHI3L1*	1	203150179	
rs7542294	*CHI3L1*	1	203151176	
rs946259	*CHI3L1*	1	203152177	
rs2071579	*CHI3L1*	1	203153634	X
rs7515776	*CHI3L1*	1	203155703	
rs4950928	*CHI3L1*	1	203155882	
rs10802971	*CHML / OPN3*	1	241739794	X
rs1053183	*CHML / OPN3*	1	241758631	
rs3753216	*CHML / OPN3*	1	241766551	X
rs616593	*CHML / OPN3*	1	241768617	
rs646355	*CHML / OPN3*	1	241768630	
rs3765813	*CHML / OPN3*	1	241772881	
rs3753219	*CHML / OPN3*	1	241775502	X
rs587640	*CHML / OPN3*	1	241790148	
rs676750	*CHML / OPN3*	1	241796284	X
rs3819981	*CHML / OPN3*	1	241800324	X
rs10184597	*IL18R1 / IL1RL1 / IL1R1*	2	102802255	X
rs3771199	*IL18R1 / IL1RL1 / IL1R1*	2	102806613	X
rs17637748	*IL18R1 / IL1RL1 / IL1R1*	2	102841715	X
rs11692230	*IL18R1 / IL1RL1 / IL1R1*	2	102855065	X
rs17689452	*IL18R1 / IL1RL1 / IL1R1*	2	102864681	X
rs12475055	*IL18R1 / IL1RL1 / IL1R1*	2	102878891	
rs12996772	*IL18R1 / IL1RL1 / IL1R1*	2	102947201	X
rs12712142	*IL18R1 / IL1RL1 / IL1R1*	2	102960584	
rs10439410	*IL18R1 / IL1RL1 / IL1R1*	2	102990788	
rs6758936	*IL18R1 / IL1RL1 / IL1R1*	2	102991369	X
rs1427680	*CTLA4*	2	204729795	X
rs11571315	*CTLA4*	2	204730901	
rs4553808	*CTLA4*	2	204731005	
rs16840252	*CTLA4*	2	204731519	
rs207908	*XRCC5*	2	217015947	X
rs3821107	*XRCC5*	2	217031328	X
rs3770507	*XRCC5*	2	217031678	
rs207939	*XRCC5*	2	217042498	X
rs207945	*XRCC5*	2	217045939	
rs2161969	*TNS1*	2	218680570	X
rs4674217	*TNS1*	2	218681575	X

rs12466972	*TNS1*	2	218683907	
rs6729330	*TNS1*	2	218694296	X
rs16858320	*TNS1*	2	218699505	
rs929936	*TNS1*	2	218709145	
rs987338	*TNS1*	2	218711738	X
rs3791978	*TNS1*	2	218714686	X
rs3791961	*TNS1*	2	218721834	X
rs2042542	*TNS1*	2	218736292	X
rs3791947	*TNS1*	2	218739125	
rs4674220	*TNS1*	2	218742002	X
rs6729299	*TNS1*	2	218750154	
rs3828282	*TNS1*	2	218779144	X
rs13022785	*TNS1*	2	218782820	X
rs4672859	*TNS1*	2	218789397	
rs3791906	*TNS1*	2	218803329	
rs7597833	*SERPINE2*	2	224842150	X
rs6721140	*SERPINE2*	2	224858643	X
rs3795877	*SERPINE2*	2	224866177	X
rs13411332	*SERPINE2*	2	224887191	
rs920251	*SERPINE2*	2	224892945	X
rs164640	*TLR9*	3	52247314	
rs352139	*TLR9*	3	52258372	X
rs352143	*TLR9*	3	52264907	
rs353547	*TLR9*	3	52268866	X
rs2284659	*SOD3*	4	24794797	X
rs699473	*SOD3*	4	24796803	
rs2536512	*SOD3*	4	24801315	
rs1799895	*SOD3*	4	24801834	
rs17552548	*SOD3*	4	24804722	
rs11944668	*FAM13A*	4	89669852	
rs2276936	*FAM13A*	4	89726283	X
rs1870339	*FAM13A*	4	89727656	
rs1379934	*FAM13A*	4	89755842	
rs7674313	*FAM13A*	4	89777061	
rs6830970	*FAM13A*	4	89777081	
rs10049947	*FAM13A*	4	89790593	X
rs1458562	*FAM13A*	4	89853598	X
rs10008568	*FAM13A*	4	89854192	
rs987314	*FAM13A*	4	89862169	X
rs2904259	*FAM13A*	4	89885714	
rs1921681	*FAM13A*	4	89904124	X
rs6844655	*FAM13A*	4	89915359	X
rs1795739	*FAM13A*	4	89930313	X
rs1398942	*FAM13A*	4	89930392	X
rs1795721	*FAM13A*	4	89943473	
rs11726569	*INTS12 / GSTCD / NPNT*	4	106606608	
rs2112047	*INTS12 / GSTCD / NPNT*	4	106698700	

rs2553434	*INTS12 / GSTCD / NPNT*	4	106798457	
rs2544425	*INTS12 / GSTCD / NPNT*	4	106801279	
rs6811135	*INTS12 / GSTCD / NPNT*	4	106830232	X
rs7677312	*INTS12 / GSTCD / NPNT*	4	106851856	
rs4635819	*INTS12 / GSTCD / NPNT*	4	106858898	
rs6817700	*INTS12 / GSTCD / NPNT*	4	106891531	X
rs1489758	*HHIP*	4	145574239	X
rs6537309	*HHIP*	4	145605075	
rs17721701	*HHIP*	4	145618719	X
rs2087826	*HHIP*	4	145639318	X
rs3763084	*PDE4D*	5	58272116	
rs7737685	*PDE4D*	5	58289046	
rs4700316	*PDE4D*	5	58294691	
rs2968005	*PDE4D*	5	58318052	X
rs1948651	*PDE4D*	5	58333340	X
rs16889129	*PDE4D*	5	58349882	
rs10061553	*PDE4D*	5	58352210	
rs6867053	*PDE4D*	5	58365545	X
rs929820	*PDE4D*	5	58393332	X
rs6898374	*PDE4D*	5	58396902	X
rs10071163	*PDE4D*	5	58409273	X
rs13186012	*PDE4D*	5	58427198	
rs13176475	*PDE4D*	5	58428799	X
rs2409629	*PDE4D*	5	58478692	X
rs8180396	*PDE4D*	5	58481760	X
rs9292197	*PDE4D*	5	58501622	X
rs11747104	*PDE4D*	5	58505337	
rs153982	*PDE4D*	5	58520536	
rs27548	*PDE4D*	5	58527071	X
rs28054	*PDE4D*	5	58531652	
rs4699939	*PDE4D*	5	58552877	X
rs26709	*PDE4D*	5	58568473	X
rs10491352	*PDE4D*	5	58573936	
rs27184	*PDE4D*	5	58575323	X
rs17780175	*PDE4D*	5	58589786	X
rs17780860	*PDE4D*	5	58623098	
rs40122	*PDE4D*	5	58628717	X
rs1823066	*PDE4D*	5	58675953	X
rs433565	*PDE4D*	5	58681898	
rs6874460	*PDE4D*	5	58701418	
rs258129	*PDE4D*	5	58711707	X
rs2547918	*PDE4D*	5	58717094	
rs17795596	*PDE4D*	5	58722921	
rs16889903	*PDE4D*	5	58782668	X
rs16889907	*PDE4D*	5	58785305	
rs1605275	*PDE4D*	5	58815363	
rs37574	*PDE4D*	5	58832869	X

rs17797781	*PDE4D*	5	58834124	X
rs40216	*PDE4D*	5	58836950	X
rs294498	*PDE4D*	5	58876950	
rs2069812	*IL5 / RAD50 / IL13 / IL4*	5	131879916	X
rs6871536	*IL5 / RAD50 / IL13 / IL4*	5	131969874	
rs2240032	*IL5 / RAD50 / IL13 / IL4*	5	131977127	
rs1800925	*IL5 / RAD50 / IL13 / IL4*	5	131992809	
rs20541	*IL5 / RAD50 / IL13 / IL4*	5	131995964	
rs848	*IL5 / RAD50 / IL13 / IL4*	5	131996500	
rs2243250	*IL5 / RAD50 / IL13 / IL4*	5	132009154	
rs2070874	*IL5 / RAD50 / IL13 / IL4*	5	132009710	
rs2243282	*IL5 / RAD50 / IL13 / IL4*	5	132016554	
rs778584	*CD14*	5	140005212	
rs2563298	*CD14*	5	140011315	
rs2569190	*CD14*	5	140012916	X
rs2569193	*CD14*	5	140015495	X
rs4357026	*SPINK5*	5	147457939	X
rs2303064	*SPINK5*	5	147480080	
rs2303065	*SPINK5*	5	147480112	
rs3777143	*SPINK5*	5	147487479	
rs1862439	*SPINK5*	5	147494931	
rs2052532	*SPINK5*	5	147496307	
rs11743440	*SPINK5*	5	147499080	X
rs7733401	*HTR4*	5	147833281	
rs6887366	*HTR4*	5	147851270	
rs4264931	*HTR4*	5	147874556	X
rs17720733	*HTR4*	5	147950478	
rs867522	*HTR4*	5	147966246	
rs2910098	*HTR4*	5	147978253	
rs2964276	*HTR4*	5	147978978	X
rs1042713	*ADRB2*	5	148206440	
rs1042714	*ADRB2*	5	148206473	X
rs1042717	*ADRB2*	5	148206646	
rs1042719	*ADRB2*	5	148207447	X
rs953569	*HAVCR1*	5	156477400	X
rs1553318	*HAVCR1*	5	156479323	X
rs6420075	*HAVCR1*	5	156486120	
rs2853694	*IL12B*	5	158749088	X
rs730691	*IL12B*	5	158756227	X
rs1736927	*HLA-G*	6	29796115	
rs1063320	*HLA-G*	6	29798749	X
rs1610696	*HLA-G*	6	29798803	
rs2523793	*HLA-G*	6	29802550	
rs2735014	*HLA-G*	6	29805809	X
rs909253	*TNFA / LTA*	6	31540313	X
rs2229094	*TNFA / LTA*	6	31540556	X
rs1041981	*TNFA / LTA*	6	31540784	

rs1800630	*TNFA / LTA*	6	31542476	
rs1800629	*TNFA / LTA*	6	31543031	
rs3093662	*TNFA / LTA*	6	31544189	
rs3093665	*TNFA / LTA*	6	31545391	
rs2269425	*AGER / PPT2*	6	32123639	
rs10947233	*AGER / PPT2*	6	32124424	
rs3134603	*AGER / PPT2*	6	32126002	
rs3134950	*AGER / PPT2*	6	32127477	
rs2269423	*AGER / PPT2*	6	32145707	X
rs3134945	*AGER / PPT2*	6	32146492	
rs9469089	*AGER / PPT2*	6	32146657	
rs3132965	*AGER / PPT2*	6	32146997	
rs3130349	*AGER / PPT2*	6	32147696	
rs3134943	*AGER / PPT2*	6	32147761	
rs1035798	*AGER / PPT2*	6	32151222	X
rs2070600	*AGER / PPT2*	6	32151443	
rs3131300	*AGER / PPT2*	6	32151934	
rs1800684	*AGER / PPT2*	6	32151994	
rs3129876	*HLADRB1 / HLADRA*	6	32408012	X
rs3129881	*HLADRB1 / HLADRA*	6	32409484	
rs9268659	*HLADRB1 / HLADRA*	6	32410941	X
rs2213585	*HLADRB1 / HLADRA*	6	32413150	
rs3129889	*HLADRB1 / HLADRA*	6	32413545	
rs9268852	*HLADRB1 / HLADRA*	6	32429594	X
rs9268877	*HLADRB1 / HLADRA*	6	32431147	
rs5020946	*HLADRB1 / HLADRA*	6	32450089	
rs9269794	*HLADRB1 / HLADRA*	6	32549249	
rs701831	*HLADRB1 / HLADRA*	6	32549395	
rs2157337	*HLADRB1 / HLADRA*	6	32609122	
rs9385992	*GPR126*	6	142633627	
rs1891308	*GPR126*	6	142648235	
rs9389986	*GPR126*	6	142661114	X
rs11155242	*GPR126*	6	142691549	
rs17071756	*GPR126*	6	142715195	
rs898070	*NPSR1*	7	34698865	X
rs1419835	*NPSR1*	7	34723920	
rs10241507	*NPSR1*	7	34743478	X
rs1419779	*NPSR1*	7	34813308	X
rs6462579	*NPSR1*	7	34866287	
rs764269	*NPSR1*	7	34875729	
rs10246825	*NPSR1*	7	34877034	X
rs10258734	*NPSR1*	7	34896123	
rs1800783	*NOS3*	7	150689397	X
rs1800779	*NOS3*	7	150689943	
rs1799983	*NOS3*	7	150696111	
rs3918227	*NOS3*	7	150700946	
rs3918188	*NOS3*	7	150702781	X

rs1808593	*NOS3*	7	150708302	
rs7830	*NOS3*	7	150709571	X
rs892248	*ANGPT1*	8	108422946	
rs1342326	*IL33*	9	6190076	
rs3939286	*IL33*	9	6210099	
rs928413	*IL33*	9	6213387	X
rs10975498	*IL33*	9	6226688	
rs2006682	*IL33*	9	6227045	X
rs10815388	*IL33*	9	6232242	X
rs7019575	*IL33*	9	6243935	X
rs10975516	*IL33*	9	6247693	X
rs1330383	*IL33*	9	6251507	
rs12000491	*IL33*	9	6257367	
rs1927914	*TLR4*	9	120464725	X
rs2770146	*TLR4*	9	120473338	X
rs5030728	*TLR4*	9	120474282	
rs11536897	*TLR4*	9	120480010	
rs485411	*GATA3*	10	8093185	
rs3802604	*GATA3*	10	8102272	X
rs376397	*GATA3*	10	8103298	
rs3802600	*GATA3*	10	8108812	
rs10905284	*GATA3*	10	8115362	X
rs573122	*MS4A2*	11	59844919	X
rs547110	*MS4A2*	11	59846718	
rs1441586	*MS4A2*	11	59856028	
rs2847667	*MS4A2*	11	59859609	X
rs2583471	*MS4A2*	11	59861814	
rs569108	*MS4A2*	11	59863104	
rs174547	*FADS1 / FADS2*	11	61570783	X
rs174548	*FADS1 / FADS2*	11	61571348	
rs968567	*FADS1 / FADS2*	11	61595564	
rs174579	*FADS1 / FADS2*	11	61605613	X
rs6591251	*GSTP1*	11	67344689	
rs762803	*GSTP1*	11	67352256	X
rs1695	*GSTP1*	11	67352689	
rs749174	*GSTP1*	11	67353253	X
rs1138272	*GSTP1*	11	67353579	
rs476391	*MMP12*	11	102735030	
rs651159	*MMP12*	11	102736419	
rs632009	*MMP12*	11	102738499	X
rs11225442	*MMP12*	11	102739319	
rs7123600	*MMP12*	11	102741968	
rs2276109	*MMP12*	11	102745791	
rs5744280	*IL18*	11	112016514	X
rs549908	*IL18*	11	112020916	X
rs5744256	*IL18*	11	112022848	
rs360722	*IL18*	11	112026703	

rs795467	*IL18*	11	112031080	
rs2043055	*IL18*	11	112031624	
rs360718	*IL18*	11	112034739	
rs757343	*VDR*	12	48239675	
rs2248098	*VDR*	12	48253356	X
rs3819545	*VDR*	12	48265006	
rs10875693	*VDR*	12	48269650	X
rs11168275	*VDR*	12	48272275	
rs2254210	*VDR*	12	48273714	X
rs2238136	*VDR*	12	48277713	X
rs4760648	*VDR*	12	48280665	X
rs11168287	*VDR*	12	48285414	X
rs4334089	*VDR*	12	48286015	X
rs4760658	*VDR*	12	48296486	X
rs3024974	*STAT6*	12	57492745	
rs841718	*STAT6*	12	57492996	
rs3024957	*STAT6*	12	57498035	
rs324011	*STAT6*	12	57502182	
rs167769	*STAT6*	12	57503775	X
rs12298170	*STAT6*	12	57515363	X
rs17119494	*STAT6*	12	57516933	
rs1732886	*IRAK3*	12	66583762	
rs1168771	*IRAK3*	12	66588836	X
rs1168774	*IRAK3*	12	66591487	
rs1168757	*IRAK3*	12	66594116	
rs1732877	*IRAK3*	12	66599144	
rs1152888	*IRAK3*	12	66605228	
rs2111059	*IFNG*	12	68541671	
rs11177072	*IFNG*	12	68542541	
rs11177073	*IFNG*	12	68542895	
rs2069718	*IFNG*	12	68550162	X
rs2069716	*IFNG*	12	68550815	
rs1861493	*IFNG*	12	68551196	
rs9658490	*NOS1*	12	117670298	
rs12830203	*NOS1*	12	117688499	X
rs11068428	*NOS1*	12	117693817	
rs4766842	*NOS1*	12	117720130	
rs733334	*NOS1*	12	117732689	X
rs7295972	*NOS1*	12	117747368	
rs1483757	*NOS1*	12	117761540	X
rs545654	*NOS1*	12	117777049	X
rs11635145	*SMAD3A*	15	67370121	X
rs7181878	*SMAD3A*	15	67389161	
rs9302242	*SMAD3A*	15	67389412	X
rs4776890	*SMAD3A*	15	67393045	X
rs7163381	*SMAD3A*	15	67414055	
rs11636161	*SMAD3A*	15	67418104	X

rs2118610	*SMAD3A*	15	67428334	X
rs745103	*SMAD3A*	15	67435075	X
rs2289263	*SMAD3A*	15	67439207	
rs10152544	*SMAD3A*	15	67444747	
rs744910	*SMAD3A*	15	67446785	X
rs7183244	*SMAD3A*	15	67461311	X
rs12708492	*SMAD3A*	15	67467541	
rs3784681	*SMAD3A*	15	67472185	X
rs3743343	*SMAD3A*	15	67486775	
rs11639224	*IREB2*	15	78753371	X
rs1964678	*IREB2*	15	78754000	X
rs8043227	*IREB2*	15	78768871	
rs647041	*CHRNA5* / *CHRNA3* / *CHRNB4*	15	78880481	
rs578776	*CHRNA5* / *CHRNA3* / *CHRNB4*	15	78888400	X
rs2869546	*CHRNA5* / *CHRNA3* / *CHRNB4*	15	78907345	
rs8042059	*CHRNA5* / *CHRNA3* / *CHRNB4*	15	78907859	
rs1878399	*CHRNA5* / *CHRNA3* / *CHRNB4*	15	78912003	X
rs11636605	*CHRNA5* / *CHRNA3* / *CHRNB4*	15	78928878	
rs2297516	*NOS2*	17	26095730	X
rs11080344	*NOS2*	17	26104511	X
rs4795067	*NOS2*	17	26106675	X
rs944725	*NOS2*	17	26109571	X
rs4795400	*ORMDL3* / *GSDMB* / *GSDMA*	17	38067020	X
rs12603332	*ORMDL3* / *GSDMB* / *GSDMA*	17	38082807	X
rs3744246	*ORMDL3* / *GSDMB* / *GSDMA*	17	38084350	
rs7207600	*ORMDL3* / *GSDMB* / *GSDMA*	17	38091660	X
rs8065126	*ORMDL3* / *GSDMB* / *GSDMA*	17	38099035	
rs7212938	*ORMDL3* / *GSDMB* / *GSDMA*	17	38122680	
rs921651	*ORMDL3* / *GSDMB* / *GSDMA*	17	38133922	
rs2241718	*TGFB1*	19	41829606	
rs4803455	*TGFB1*	19	41851509	X
rs1800470	*TGFB1*	19	41858921	
rs1800469	*TGFB1*	19	41860296	
rs2317130	*TGFB1*	19	41861674	X
rs2241713	*TGFB1*	19	41869468	X
rs677044	*ADAM33*	20	3649431	
rs2280091	*ADAM33*	20	3650234	
rs2853209	*ADAM33*	20	3651472	
rs3918395	*ADAM33*	20	3653149	
rs511898	*ADAM33*	20	3655085	X
rs3918392	*ADAM33*	20	3655219	
rs2853210	*ADAM33*	20	3658211	X
rs487377	*ADAM33*	20	3658931	
rs570269	*ADAM33*	20	3659647	
rs554743	*ADAM33*	20	3662142	
rs3918249	*MMP9*	20	44638136	
rs2274755	*MMP9*	20	44639692	

rs17576	*MMP9*	20	44640225	X
rs2236416	*MMP9*	20	44640575	
rs9607267	*HMOX1*	22	35781207	
rs11912889	*HMOX1*	22	35783617	
rs84460	*IL2RB*	22	37525731	X
rs228945	*IL2RB*	22	37525880	
rs2072861	*IL2RB*	22	37528362	
rs2072862	*IL2RB*	22	37528606	X
rs228963	*IL2RB*	22	37535948	
rs228966	*IL2RB*	22	37537514	X
rs3218264	*IL2RB*	22	37541998	
rs3218258	*IL2RB*	22	37544245	X

SNP: single nucleotide polymorphism; GEIRD: Gene Environment Interactions in Respiratory Diseases; Chr Pos: Chromosome Position.

Paragraph S1. Gene selection in the GEIRD study.

The gene selection was based on their association with asthma, COPD, or allergic rhinitis, from the literature (full reference list is reported in **Table S2**), or on their involvement in possible related biological pathways (e.g. inflammation, innate immunity and immune-regulation, oxidative stress metabolism, tissue remodelling). SNPs in these gene or gene regions included both SNPs tagging most of haplotype variability in the CEU population (HapMAp phase II) and SNPs from literature (*www.ncbi.nlm.nih.gov/snp*). STAMPA application (GEVALT software; *acgt.cs.tau.ac.il/gevalt/#ver2*) was used for the selection of these SNPs, which constituted the optimal set of tag-SNPs representative of a given genomic region with high LD and maximum prediction accuracy.

Table S2. List of the references that were used to select the 53 genes or gene regions in the GEIRD survey (2008-2010).

Allen, M.; Heinzmann, A.; Noguchi, E.; Abecasis, G.; Broxholme, J.; Ponting, C.P.; Bhattacharyya, S.; Tinsley, J.; Zhang, Y.; Holt, R.; et al. Positional cloning of a novel gene influencing asthma from chromosome 2q14. *Nat Genet* **2003,** *35*, 258-63.

Aynacioglu, A.S.; Nacak, M.; Filiz, A.; Ekinci, E.; Roots, I. Protective role of glutathione S-transferase P1 (GSTP1) Val105Val genotype in patients with bronchial asthma. *Br J Clin Pharmacol* **2004,** *57*, 213-7.

Brasch-Andersen, C.; Christiansen, L.; Tan, Q.H.; Haagerup, A.; Vestbo, J.; Kruse, T.A. Possible gene dosage effect of glutathione-S-transferases on atopic asthma: Using real-time PCR for quantification of GSTM1 and GSTT1 gene copy numbers. *Hum Mutat* **2004,** *24*, 208-214.

Carlson, C.S.; Eberle, M.A.; Kruglyak, L.; Nickerson, D.A. Mapping complex disease loci in whole-genome association studies. *Nature* **2004,** *429*, 446-452.

Chung, K.F. Inflammatory mediators in chronic obstructive pulmonary disease. *Curr Drug Targets Inflamm Allergy* **2005,** *4*, 619-25.

Contopoulos-Ioannidis, D.G.; Kouri, I.N.; Ioannidis, J.P. Genetic predisposition to asthma and atopy. *Respiration* **2007,** *74*, 8-12.

Cookson, W. Genetics and genomics of asthma and allergic diseases. *Immunol Rev* **2002,** *190*, 195-206.

Cookson, W. The immunogenetics of asthma and eczema: a new focus on the epithelium. *Nat Rev Immunol* **2004,** *4*, 978-88.

Fireman, P. Cytokines and allergic rhinitis. *Allergy Asthma Proc* **1996,** *17*, 175-8.

Gohlke, H.; Illig, T.; Bahnweg, M.; Klopp, N.; Andre, E.; Altmuller, J.; Herbon, N.; Werner, M.; Knapp, M.; Pescollderungg, L.; et al. Association of the interleukin-1 receptor antagonist gene with asthma. *Am J Respir Crit Care Med* **2004,** *169*, 1217-23.

Hakonarson, H.; Wjst, M. Current concepts on the genetics of asthma. *Curr Opin Pediatr* **2001,** *13*, 267-77.

Hawkins, G.A.; Weiss, S.T.; Bleecker, E.R. Clinical consequences of ADRbeta2 polymorphisms. *Pharmacogenomics* **2008,** *9*, 349-58.

Hoh, J.; Ott, J. Genetic dissection of diseases: design and methods. *Curr Opin Genet Dev* **2004,** *14*, 229-32.

Jongepier, H.; Boezen, H.M.; Dijkstra, A.; Howard, T.D.; Vonk, J.M.; Koppelman, G.H.; Zheng, S.L.; Meyers, D.A.; Bleecker, E.R.; Postma, D.S. Polymorphisms of the ADAM33 gene are associated with accelerated lung function decline in asthma. *Clin Exp Allergy* **2004,** *34*, 757-60.

Kere, J.; Laitinen, T. Positionally cloned susceptibility genes in allergy and asthma. *Curr Opin Immunol* **2004,** *16*, 689-94.

Kim, W.J.; Oh, Y.M.; Sung, J.; Lee, Y.K.; Seo, J.B.; Kim, N.; Kim, T.H.; Huh, J.W.; Lee, J.H.; Kim, E.K.; et al. CT scanning-based phenotypes vary with ADRB2 polymorphisms in chronic obstructive pulmonary disease. *Respir Med* **2009,** *103*, 98-103.

Malerba, G.; Pignatti, P.F. A review of asthma genetics: gene expression studies and recent candidates. *J Appl Genet* **2005,** *46*, 93-104.

Martinez, F.D. Gene-Environment Interaction in Complex Diseases: Asthma as an Illustrative Case. *Novartis Found Symp* **2008**, *293*, 184-92; discussion 192-7.

Meyers, D.A.; Larj, M.J.; Lange, L. Genetics of asthma and COPD. Similar results for different phenotypes. *Chest* **2004,** *126*, 105S-110S; discussion 159S-161S.

Moffatt, M.F.; Faux, J.A.; Lester, S.; Pare, P.; McCluskey, J.; Spargo, R.; James, A.; Musk, A.W.; Cookson, W.O.C.M. Atopy, respiratory function and HLA-DR in aboriginal Australians. *Human Molecular Genetics* **2003,** *12*, 625-630.

Raby, B.A.; Silverman, E.K.; Lazarus, R.; Lange, C.; Kwiatkowski, D.J.; Weiss, S.T. Chromosome 12q harbors multiple genetic loci related to asthma and asthma-related phenotypes. *Hum Mol Genet* **2003,** *12*, 1973-9.

Recalde, H.; Cuccia, M.; Oggionni, T.; Dondi, E.; Martinetti, M.; Luisetti, M. Lymphocyte expression of human leukocyte antigen class II molecules in patients with chronic obstructive pulmonary disease. *Monaldi Arch Chest Dis* **1999,** *54*, 384-9.

Rimmer, J.; Peake, H.L.; Santos, C.M.; Lean, M.; Bardin, P.; Robson, R.; Haumann, B.; Loehrer, F.; Handel, M.L. Targeting adenosine receptors in the treatment of allergic rhinitis: a randomized, double-blind, placebo-controlled study. *Clin Exp Allergy* **2007,** *37*, 8-14.

Shiina, T.; Inoko, H.; Kulski, J.K. An update of the HLA genomic region, locus information and disease associations: 2004. *Tissue Antigens* **2004,** *64*, 631-49.

Tamer, L.; Calikoglu, M.; Ates, N.A.; Yildirim, H.; Ercan, B.; Saritas, E.; Unlu, A.; Atik, U. Glutathione-S-transferase gene polymorphisms (GSTT1, GSTM1, GSTP1) as increased risk factors for asthma. *Respirology* **2004,** *9*, 493-498.

Van Eerdewegh, P.; Little, R.D.; Dupuis, J.; Del Mastro, R.G.; Falls, K.; Simon, J.; Torrey, D.; Pandit, S.; McKenny, J.; Braunschweiger, K.; et al. Association of the ADAM33 gene with asthma and bronchial hyperresponsiveness. *Nature* **2002,** *418*, 426-30.

Vercelli, D. Discovering susceptibility genes for asthma and allergy. *Nat Rev Immunol* **2008,** *8*, 169-82.

Vercelli, D. Gene-environment interactions: the road less traveled by in asthma genetics. *J Allergy Clin Immunol* **2009,** *123*, 26-7.

von Mutius, E. Gene-environment interactions in asthma. *J Allergy Clin Immunol* **2009,** *123*, 3-11; quiz 12-3.

Wright, R.J. Make no bones about it - Increasing epidemiologic evidence links vitamin D to pulmonary function and COPD. *Chest* **2005,** *128*, 3781-3783.

Wjst, M.; Fischer, G.; Immervoll, T.; Jung, M.; Saar, K.; Rueschendorf, F.; Reis, A.; Ulbrecht, M.; Gomolka, M.; Weiss, E.H.; et al. A genome-wide search for linkage to asthma. German Asthma Genetics Group. *Genomics* **1999,** *58*, 1-8.

Zhang, J.; Pare, P.D.; Sandford, A.J. Recent advances in asthma genetics. *Respir Res* **2008,** *9*, 4.

Paragraph S2. Definition of asthma in the ECRHS II study.

An asthma case was a subject who fulfilled at least one of the following criteria:

- ever asthma OR (asthma attacks/asthma-like symptoms/anti-asthmatic drugs in the previous 12 months AND PD_{20} <1 mg) OR (asthma attacks/asthma-like symptoms/anti-asthmatic drugs in the previous 12 months AND pre-BD FEV_1/FVC <LLN [Quanjer, 2012] or <70%) at ECRHS I;
- ever asthma OR (asthma attacks/asthma-like symptoms/anti-asthmatic drugs in the previous 12 months AND PD_{20} <1 mg) OR (asthma attacks/asthma-like symptoms/anti-asthmatic drugs in the previous 12 months AND pre-BD FEV_1/FVC <LLN or <70%) at ECRHS II.

Quanjer, P.H.; Stanojevic, S.; Cole, T.J.; Baur, X.; Hall, G.L.; Culver, B.H.; Enright, P.L.; Hankinson, J.L.; Ip, M.S.; Zheng, J.; *et al*. Multi-ethnic reference values for spirometry for the 3-95-yr age range: the global lung function 2012 equations. *Eur Respir J* **2012,** *40* (6), 1324-43. https://doi.org/10.1183/09031936.00080312

Table S3. Main characteristics of the asthma cases according to their inclusion in the genetic association analysis (GEIRD survey).

		Included	Excluded[a]	P-value[b]
Sample, n		143	243	-
Females, %		49.7	51.0	0.794
Age (years), median (IQR)		42.1 (34.9, 48.5)	43.8 (36.8, 49.8)	0.098
BMI, median (IQR)		24.5 (22.0, 27.1)	24.1 (21.9, 26.9)	0.634
Tobacco smoking, %	Never	48.9	46.1	0.419
	Past	22.4	28.4	
	Current	28.7	25.5	
Nasal allergies, %	Absent	30.1	53.1	<0.001
	Present	69.2	46.1	
	Missing	0.7	0.8	
Eczema/Skin allergies, %		34.3	32.8	0.765
Itchy rash, %		14.0	11.6	0.498
Pre-BD FEV_1 % predicted, median (IQR)		95.5 (85.7, 108.3)	97.5 (87.6, 106.8)	0.733
Pre-BD FVC % predicted, median (IQR)		101.7 (92.2, 110.0)	99.0 (88.1, 110.2)	0.359
Pre-BD FEV_1/FVC % predicted, median (IQR)		93.3 (89.4, 100.4)	93.6 (87.8, 101.2)	0.643
Total IgE (kU/L), geometric mean (95%CI)		111.5 (91.5, 135.8)	66.2 (52.3, 83.8)	0.036

GEIRD: Gene Environment Interactions in Respiratory Diseases; IQR: interquartile range; BMI: body mass index; pre-BD: pre-bronchodilator; FEV_1: forced expiratory volume in one second; FVC: forced vital capacity; IgE: Immunoglobulin E; kU/L: kiloUnits per Litre; 95%CI: 95% confidence interval. [a]Asthma cases who were excluded from the genetic association analysis because they had not been genotyped (44 subjects) or, among those with genetic data, because (i) they lacked information on total IgE or (ii) they had reported no respiratory symptoms in presence of perennial and seasonal allergens or (iii) they had reported respiratory symptoms in presence of seasonal allergens only (199 subjects). [b]Pearson chi-squared test, Fisher's exact test, Wilcoxon rank-sum test, or likelihood-ratio test were used when needed.

Paragraph S3. Supplementary information on ECRHS III

Steering Committee: U. Ackermann-Liebrich (University of Basel, Switzerland); N. Kuenzli (University of Basel, and University of Southern California, Los Angeles, USA); J.M. Antó and J. Sunyer (Institut Municipal d' Investigació Médica (IMIM-IMAS), Universitat Pompeu Fabra (UPF), Spain); P. Burney (project leader), S Chinn, D. Jarvis, J. Knox and C. Luczynska (King's College London, UK); I. Cerveri (University of Pavia, Italy); R. de Marco† (University of Verona, Italy); T. Gislason (Iceland University Hospital, Iceland); J. Heinrich and M. Wjst (GSF–Institute of Epidemiology, Germany); C. Janson (Uppsala University, Sweden); B. Leynaert and F. Neukirch (Institut National de la Santé et de la Recherche Medicale (INSERM), France); J. Schouten (University of Groningen, The Netherlands); C. Svanes (University of Bergen, Norway); P. Vermeire† (University of Antwerp, Belgium).

† deceased

Principal Investigators and senior scientific teams: **Australia: Melbourne** (M. Abramson, E.H. Walters, J. Raven); **Belgium: South Antwerp and Antwerp City** (P. Vermeire, J. Weyler, M. van Sprundel, V. Nelen); **Estonia: Tartu** (R. Jõgi, A. Soon); **France: Paris** (F. Neukirch, B. Leynaert, R. Liard, M. Zureik), **Grenoble** (I. Pin, J. Ferran-Quentin), **Bordeaux** (A. Taytard, C. Raherison), **Montpellier** (J. Bousquet, P.J. Bousquet); **Germany: Erfurt** (J. Heinrich, M. Wjst, C. Frye, I. Meyer); **Iceland: Reykjavik** (T. Gislason, E. Bjornsson, D. Gislason, K.B. *Jörundsdóttir*); **Italy: Turin** (R. Bono, M. Bugiani, P. Piccioni, E. Caria, A. Carosso, E. Migliore, G. Castiglioni), **Verona** (R. de Marco†, G. Verlato, E. Zanolin, S. Accordini, A. Poli, V. Lo Cascio, M. Ferrari, I. Cazzoletti), **Pavia** (A. Marinoni, S. Villani, M. Ponzio, F. Frigerio, M. Comelli, M. Grassi, I. Cerveri, A. Corsico); **Norway: Bergen** (A. Gulsvik, E. Omenaas, C. Svanes, B. Laerum); **Spain: Albacete** (J. Martinez-Moratalla Rovira, E. Almar, M. Arévalo, C. Boix, G. González, J.M. Ignacio García, J. Solera, J. Damián), **Galdakao** (N. Muñozguren, J. Ramos, I. Urrutia, U. Aguirre), **Barcelona** (J.M. Antó, J. Sunyer, M. Kogevinas, J.P. Zock, X. Basagaña, A. Jaen, F. Burgos, C. Acosta), **Huelva** (J. Maldonado, A. Pereira, J.L. Sanchez), **Oviedo** (F. Payo, I. Huerta, A. de la Vega, L. Palenciano, J. Azofra, A. Cañada); **Sweden**: **Göteborg** (K. Toren, L. Lillienberg, A.C. Olin, B. Balder, A. Pfeifer-Nilsson, R. Sundberg), **Umea** (E. Norrman, M. Soderberg, K.A. Franklin, B. Lundback, B. Forsberg, L. Nystrom), **Uppsala** (C. Janson, G. Boman, D. Norback, G. Wieslander, M. Gunnbjornsdottir); **Switzerland: Basel** (N. Küenzli, B. Dibbert, M. Hazenkamp, M. Brutsche, U. Ackermann-Liebrich); **United Kingdom: Ipswich** (D. Jarvis, R. Hall, D. Seaton), **Norwich** (D. Jarvis, B. Harrison).

Financial Support: **Australia:** National Health and Medical Research Council; **Belgium: Antwerp:** Fund for Scientific Research (G.0402.00), University of Antwerp, Flemish Health Ministry; **Estonia: Tartu:** Estonian Science Foundation grant no 4350; **France: (All)** Programme Hospitalier de Recherche Clinique—Direction de la Recherche Clinique (DRC) de Grenoble 2000 number 2610, Ministry of Health, Ministère de l'Emploi et de la Solidarité, Direction Génerale de la Santé, Centre Hospitalier Universitaire (CHU) de Grenoble; **Bordeaux:** Institut Pneumologique d'Aquitaine; **Grenoble:** Comité des Maladies Respiratoires de l'Isere; **Montpellier:** Aventis (France), Direction Regionale des Affaires Sanitaires et Sociales Languedoc-Roussillon; **Paris:** Union Chimique Belge-Pharma (France), Aventis (France), Glaxo France; **Germany: Erfurt:** GSF—National Research Centre for Environment and Health, Deutsche Forschungsgemeinschaft (FR1526/1-1); **Hamburg:** GSF—National Research Centre for Environment and Health, Deutsche Forschungsgemeinschaft (MA 711/4-1); **Iceland: Reykjavik:** Icelandic Research Council, Icelandic University Hospital Fund; **Italy: Pavia:** GlaxoSmithKline Italy, Italian Ministry of University and Scientific and Technological Research (MURST), Local University Funding for Research 1998 and 1999; **Turin:** Azienda Sanitaria Locale 4 Regione Piemonte (Italy), Azienda Ospedaliera Centro Traumatologico Ospedaliero/Centro Traumatologico Ortopedico—Istituto Clinico Ortopedico Regina Maria Adelaide Regione Piemonte; **Verona:** Ministero dell'Università e della Ricerca Scientifica (MURST), Glaxo Wellcome spa; **Norway: Bergen:** Norwegian Research Council, Norwegian Asthma and Allergy Association, Glaxo Wellcome AS, Norway Research Fund; **Spain: Albacete:** Fondo de Investigaciones Sanitarias (97/0035-01, 99/0034-01, and 99/0034-02), Hospital Universitario de Albacete, Consejeria de Sanidad; **Barcelona:**

Sociedad Espanola de Neumología y Cirugía Toracica, Public Health Service (R01 HL62633-01), Fondo de Investigaciones Sanitarias (97/0035-01, 99/0034-01, and 99/0034-02), Consell Interdepartamental de Recerca i Innovació Tecnològica (1999SGR 00241), Instituto de Salud Carlos III, Red de Centros de Epidemiología y Salud Pública (C03/09), Red de Bases moleculares y fisiológicas de las Enfermedades Respiratorias (C03/011), Red de Grupos Infancia y Medio Ambiente (G03/176); **Huelva:** Fondo de Investigaciones Sanitarias (97/0035-01, 99/0034-01, and 99/0034-02); **Galdakao:** Basque Health Department; **Oviedo:** Fondo de Investigaciones Sanitarias (97/0035-02, 97/0035, 99/0034-01, 99/0034-02, 99/0034-04, 99/0034-06, 99/350, and 99/0034-07), European Commission (EU-PEAL PL01237), Generalitat de Catalunya (CIRIT 1999 SGR 00214), Hospital Universitario de Albacete, Sociedad Española de Neumología y Cirugía Torácica (SEPAR R01 HL62633-01), Red de Centros de Epidemiología y Salud Pública (C03/09), Red de Bases moleculares y fisiológicas de las Enfermedades Respiratorias (C03/011), Red de Grupos Infancia y Medio Ambiente (G03/176, 97/0035-01, 99/0034-01, and 99/0034-02); **Sweden: Göteborg, Umea, and Uppsala**: Swedish Heart Lung Foundation, Swedish Foundation for Health Care Sciences and Allergy Research, Swedish Asthma and Allergy Foundation, Swedish Cancer and Allergy Foundation, Swedish Council for Working Life and Social Research (FAS); **Switzerland: Basel:** Swiss National Science Foundation, Swiss Federal Office for Education and Science, Swiss National Accident Insurance Fund; **UK: Ipswich and Norwich**: Asthma UK (formerly known as National Asthma Campaign).

Coordination: The coordination of this work was supported by the European Commission, as part of their Quality of Life programme (QLK4-CT-1999-01237).
